# Comparative Study on Cytological Characteristics of Reproductive Organs and Embryonic Development in Three Forms of the Mycoheterotrophic Orchid *Gastrodia elata*

**DOI:** 10.3390/plants15081277

**Published:** 2026-04-21

**Authors:** Haixin Diao, Shunxing Guo

**Affiliations:** StateKey Laboratory of Bioactive Substance and Function of Natural Medicines, Institute of Medicinal Plant Development, Chinese Academy of Medical Sciences/Peking Union Medical College, Beijing 100193, China; dhxtx1216@163.com

**Keywords:** *Gastrodia elata*, form, reproductive development, pollen wall polarity, nuclear clearance, programmed cell death, embryonic development, seed coat differentiation

## Abstract

*Gastrodia elata* is a mycoheterotrophic orchid. Three of its forms (*G. elata* f. *glauca*, *G. elata* f. *elata*, *G. elata* f. *viridis*) show distinct reproductive traits, but the cytological basis remains unclear. Using multi-timepoint morphological observation and semi-thin/ultra-thin sectioning, we systematically compared their reproductive processes from gametophyte development to seed maturation. All forms exhibited pollen wall polar differentiation (“well-developed locular side, simplified lateral sides”) following a six-stage program, with f*. elata* germinating fastest, f.* viridis* intermediate, and no germination in f. *glauca*. In the female gametophyte, vesicle clusters and degradation zones suggest the possibility of a two-step “chalazal degradation—micropylar localization” nuclear clearance model. Embryo development rate followed f. *elata* > f. *glauca* > f.* viridis*. Mature seeds stored lipid/protein bodies; f*. elata* uniquely contained amyloplasts and acicular phytin crystals, with form-specific seed coat traits. This study clarifies cytological differentiation, providing a basis for germplasm identification and conservation.

## 1. Introduction

### 1.1. Specialization of Reproductive Development in Orchids and Uniqueness of Saprophytic Groups

Orchidaceae is one of the most species-rich plant families, with approximately 28,000 species worldwide. Its reproductive development has evolved specialized traits such as pollinia, gynostemia, ovules without integument tapetum, and non-endospermic seeds [1,2], which are closely associated with entomophily and fungal symbiosis [3]. Mycoheterotrophic orchids have lost photosynthetic capacity and rely entirely on fungi for carbon and nutrients, potentially evolving adaptive strategies such as simplified pollen structures and enhanced fungal dependence for seed germination [4]. Approximately 23 genera and 81 species of mycoheterotrophic orchids are distributed in China, mainly belonging to *Gastrodia*, *Neottia*, and other genera [5]. Their mycorrhizal fungi are dominated by ectomycorrhizal fungi (ECM) and non-rhizoctonia saprotrophic fungi, which differ significantly from those of photosynthetic orchids [5]. For example, seed germination of *G. elata* depends on fungi of the genus *Mycena*, while its vegetative growth is symbiotic with *Armillaria* sp. [6]. However, specialized reproductive structures and symbiotic dependence make mycoheterotrophic orchids highly sensitive to environmental changes, with approximately 30% of orchid species endangered globally [7]. Elucidating the cytological characteristics of reproductive development in mycoheterotrophic orchids is of great significance for both plant reproductive evolution theory and the conservation of endangered orchid species.

### 1.2. Biological Characteristics, Cultivation Status, and Research Basis of G. elata

*G. elata* is a perennial mycoheterotrophic orchid without roots or leaves, and its entire life cycle relies on fungal symbiosis [4,8]. Its dried tubers contain bioactive components such as gastrodin, making it a precious traditional Chinese medicinal herb with large-scale artificial cultivation [9]. *G. elata* is a hermaphroditic plant with racemose inflorescences; its pollinia are pollinated by halictid bees, with a wild fruiting rate of only about 20% [10,11], and its seeds are tiny and non-endospermic, with extremely low natural germination rates [12]. Due to overharvesting and habitat fragmentation, wild *G. elata* has been listed in the National Key Protected Wild Plant List [13]. Artificial cultivation also faces problems such as germplasm degradation and unstable yield and quality [14], with the simplification of sexual reproductive structures and low reproductive efficiency being one of the core limiting factors [10].

*G. elata* f. *glauca*, *G. elata* f. *elata*, and *G. elata* f. *viridis* are three core industrial germplasms [6], showing significant differences in growth vigor, flowering phenology, tuber quality, and seed viability [15,16,17]. Their natural vertical distribution is also distinctly differentiated [6]: f.* elata* is mostly distributed at altitudes of 500–1500 m, f. *glauca* mainly at 1400–2800 m, and f. *viridis* often mixed with f. *glauca* or distributed in middle-altitude areas. However, the cytological basis of these differences remains unclear. Early studies have initially established the basic framework of embryonic development in *G. elata*: Liang [18,19] described microsporogenesis, megasporogenesis, gametophyte development, and embryogenesis of f.* glauca* using paraffin sectioning; Zhou et al. [10] systematically elaborated on the morphology and embryology of *G. elata*; Li et al. [20] analyzed the embryogenic process of f. *elata* using semi-thin sectioning. Restricted by technical conditions, however, there are still many gaps at the ultrastructural level, making it difficult to reveal fine regulatory strategies and inter-form differentiation characteristics.

### 1.3. Research Gaps in Reproductive Development of G. elata

Existing studies have several key limitations: (1) The complete developmental program and polar differentiation characteristics of pollen wall development lack systematic analysis. Pollen wall formation in orchids relies on the synergistic logic of “sporophyte supply—gametophyte template” [21], but as a mycoheterotrophic group, whether the pollen wall of *G. elata* has simplified characteristics different from those of photosynthetic orchids, and the inter-form differences in sporopollenin deposition and pollen tube germination, remain unclear, while pollen wall structure directly affects pollen viability and fertilization success [22]. (2) The ultrastructural characteristics of the degeneration of the two chalazal nuclei in the female gametophyte and the transport pathway of their debris have not been revealed. Classic light microscopy studies only confirmed the degeneration of these two nuclei before embryo sac maturation [19,20], but the fate of the debris is unknown, making it difficult to clarify the spatial isolation between functional compartments and degenerative remnants. (3) The timeline, ultrastructural characteristics, and inter-form differences in programmed cell death (PCD) in maternal tissues (nucellar epidermal cells, integument cells) after fertilization have not been reported. In angiosperms, PCD of nucellar tissue provides space for embryo sac expansion, and degradation of integument cells transports nutrients to the embryo [23,24], but the fine timeline and inter-form differentiation of this process in *G. elata* have not been resolved. (4) There is insufficient cytological evidence for the differentiation of embryonic development rhythm, seed storage substances, and seed coat structure among the three forms. The number of embryonic cells and embryo volume of orchid seeds are positively correlated with germination potential [25,26], but direct cytological evidence is lacking for whether diverse reproductive investment strategies exist among *G. elata* forms and their correlation with altitude adaptation. These gaps limit the understanding of the reproductive evolution of mycoheterotrophic orchids and the optimization of *G. elata* germplasm evaluation, breeding, and cultivation.

### 1.4. Research Objectives and Significance

This study took *G. elata* f.* glauca*, *G. elata* f. *elata*, and *G. elata* f.* viridis* as research objects, combining morphological observation, semi-thin/ultra-thin sectioning, and statistical analysis to systematically explore the dynamic cytological characteristics of their reproductive development. Specific objectives include: clarifying the complete developmental program and polar differentiation of pollen wall development and elucidating inter-form differences in pollen structure and germination; analyzing the ultrastructural characteristics of the degeneration of the two chalazal nuclei in the female gametophyte and the debris transport process; recording the timeline and ultrastructural characteristics of PCD in maternal tissues and suspensor cells; identifying the differentiation characteristics of embryonic development rhythm, seed storage substances, and seed coat structure among the three forms, and discussing their potential correlation with altitude adaptation. The results will provide theoretical support for *G. elata* germplasm identification, breeding, and cultivation optimization, and enrich the cytological theory of reproductive development in mycoheterotrophic orchids.

## 2. Results

### 2.1. Morphological and Phenological Characteristics of Flowers and Capsules

**Figure A1:**
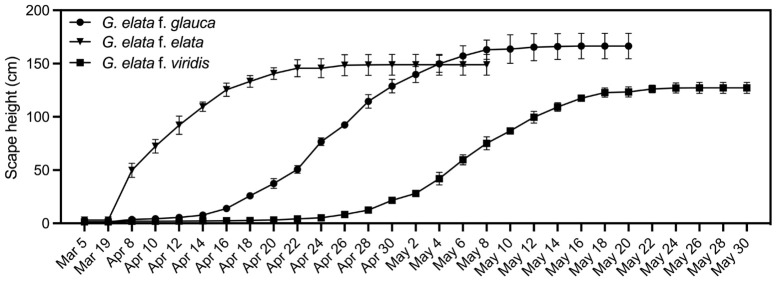
Scape growth dynamics in three *G. elata* forms (*n* = 15).

Significant inter-form differences were observed in the morphological and phenological characteristics of flowers and capsules (Table A1 & Figure A1). Among floral traits, f.* elata* showed the most vigorous vegetative growth, with a scape growth rate (2.34 ± 0.22 cm/day) and flower number per plant (88 ± 21) significantly higher than those of f.* glauca* and f.* viridis*, while f. *viridis* had the lowest scape growth rate (0.96 ± 0.05 cm/day). In terms of phenology, f. *elata* flowered earliest (12 April) with the longest flowering period (12 ± 2 d), followed by f. *glauca* (26 April, 9 ± 2 d) and f. *viridis* (1 May, 10 ± 2 d). The perianth tube of f.* elata* was the longest (11.45 ± 0.48 mm) and widest (7.8 ± 0.22 mm), while that of f.* viridis* was the smallest. The ovary expansion rate was highest in f. *elata* (0.24 ± 0.02 mm/day) and lowest in f.* viridis* (0.19 ± 0.02 mm/day).

**Figure 1:**
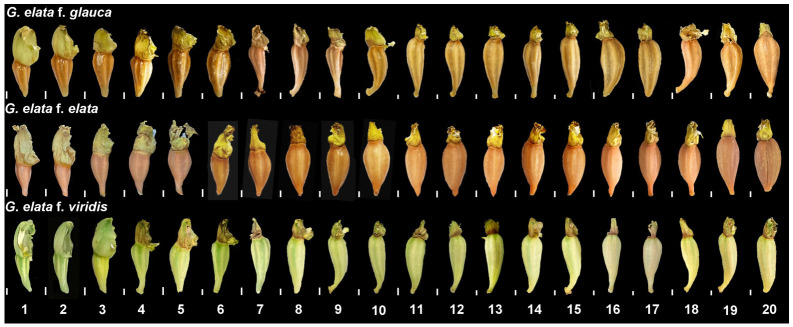
Capsule growth dynamics in three *G. elata* forms from 1 to 20 days after pollination (DAP). Scale bar = 2 mm.

Regarding capsule traits, there was no significant difference in fruiting period duration among the three forms (Table A1 & Figure 1), but the fruit maturation time of f.* glauca* and f.* viridis* was 14 d and 18 d later than that of f.* elata*, respectively. No significant difference was found in capsule width; the capsule length and length-to-width ratio of f. *glauca* and f.* viridis* were significantly higher than those of f.* elata*. f.* viridis* had the largest single fruit weight (0.44 ± 0.03 g), while f.* elata* produced fruit with the least weight (0.32 ± 0.03 g). The seed viability of f.* elata* was the highest (97.39 ± 2.96%), significantly higher than that of either f. *viridis* (86.04 ± 5.64%) or f.* glauca* (56.54 ± 4.92%).

### 2.2. Morphological Staging of Anther Development and Pollination Process

**Figure 2:**
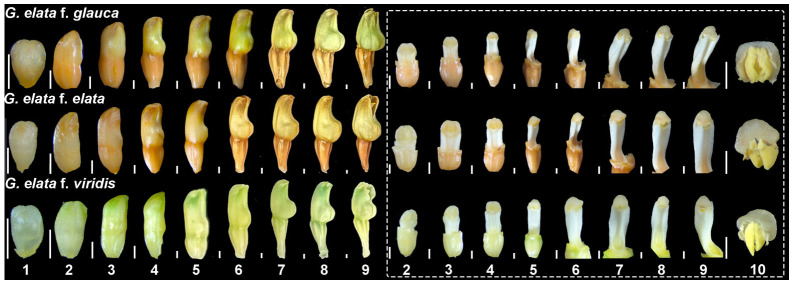
Floral bud differentiation to anthesis and corresponding anther morphology in three *G. elata* forms. Numbers 1–10 indicate developmental stages: 1 = Stage 1 (meristematic stage); 2 = Stage 2 (differentiation stage); 3 = Stage 3 (elongation stage); 4–10 = Stage 4 (separation stage). Dashed boxes show anther morphology corresponding to each floral stage. Scale bar = 1 mm.

Based on external morphological characteristics (Figure 2), the entire process of anther development from differentiation to pollination response was divided into five consecutive stages: Stage 1 (Meristematic stage): Anther and ovary differentiation were not obvious, completely enclosed by bracts; Stage 2 (Differentiation stage): Bracts and petals were detachable, with transparent calyptra and bilobed anthers visible; Stage 3 (Elongation stage): Gynostemium elongated significantly, and calyptra tissue thickened to cover the anther apex; Stage 4 (Separation stage): Calyptra shrank due to water loss and separated from the anther, and pollination was possible after full anthesis; Stage 5 (Germination stage): Pollen tubes germinated and completed fertilization after anther contact with the stigma.

### 2.3. Staging of Seed and Embryo Development

**Figure 3:**
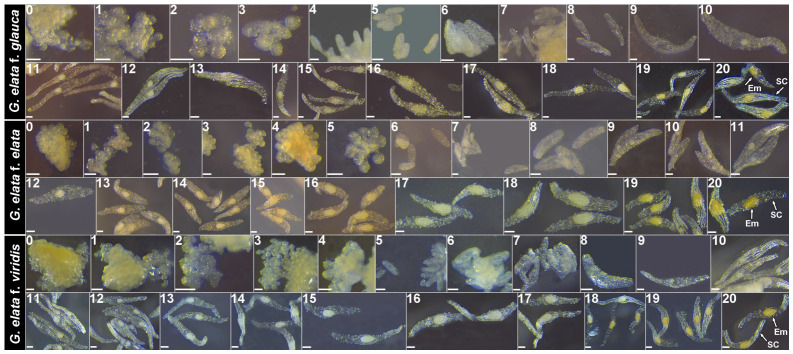
Morphological development of seeds and embryos in three *G. elata* forms (0–20 DAP). Numbers (0–20) indicate DAP. Abbreviations: Em = embryo; SC = seed coat. Scale bar = 100 µm.

According to the morphology (Figure 3) at 0–20 DAP, the entire process of seed and embryonic development was divided into six consecutive stages, with obvious developmental asynchrony among the forms: f.* glauca* entered each stage earliest, followed by f. *elata*, and then f. *viridis*.

**Figure A2:**
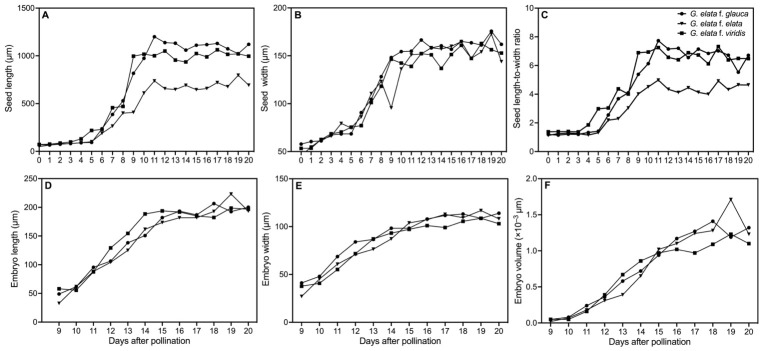
Dynamics of seed and embryo size in three *G. elata* forms from 0 to 20 DAP (*n* = 15). (**A**) Seed length. (**B**) Seed width. (**C**) Seed length-to-width ratio. (**D**) Embryo length. (**E**) Embryo width. (**F**) Embryo volume.

Based on the growth dynamics (Table 1 & Figure A2), these six stages were clearly distinguished by morphological transition points and growth rate inflection points. Stage I (Spherical stage): Seeds were spherical, with growth in the lag phase; Stage II (Obovate stage): Seeds turned obovate, seed growth entered the exponential phase in f. *viridis* and f.* elata* but remained in the lag phase in f.* glauca*, and embryo growth was consistently delayed in all forms; Stage III (Elliptical stage): Seeds became elliptical, seed growth entered the exponential phase, and embryo growth remained stagnant. Stage IV (Fusiform initiation stage): Seeds were fusiform, embryo growth initiated (visible under a stereomicroscope), seed growth maintained the exponential phase in f.* elata* and f.* glauca*, and entered the transition phase in f. *viridis*; Stage V (Embryo exponential growth stage): Embryos were obovate, and seed and embryo growth transitioned to the stationary phase; Stage VI (Maturation stage): Seed and embryo growth reached the stationary phase in all forms. Significant differences were found in the size of mature seeds and embryos among the three forms (Figure A2): seeds of f.* glauca* and f*. viridis* were slender, with a length approximately 1.28 times that of f.* elata*, but f. *elata* had the largest embryo volume (1.44 times that of f. *glauca* and 1.40 times that of f.* viridis*).

### 2.4. Microsporogenesis, Male Gametophyte Development, and Germination Response

#### 2.4.1. Inter-Form Specificity in Meiosis and Early Development

Microsporogenesis and male gametophyte development in the three forms all followed the typical process: sporogenous cell differentiation → microspore mother cell meiosis → tetrad formation → microspore mitosis → bicellular pollen, but asynchronous anther sac development was common (Table 2).

**Figure 4:**
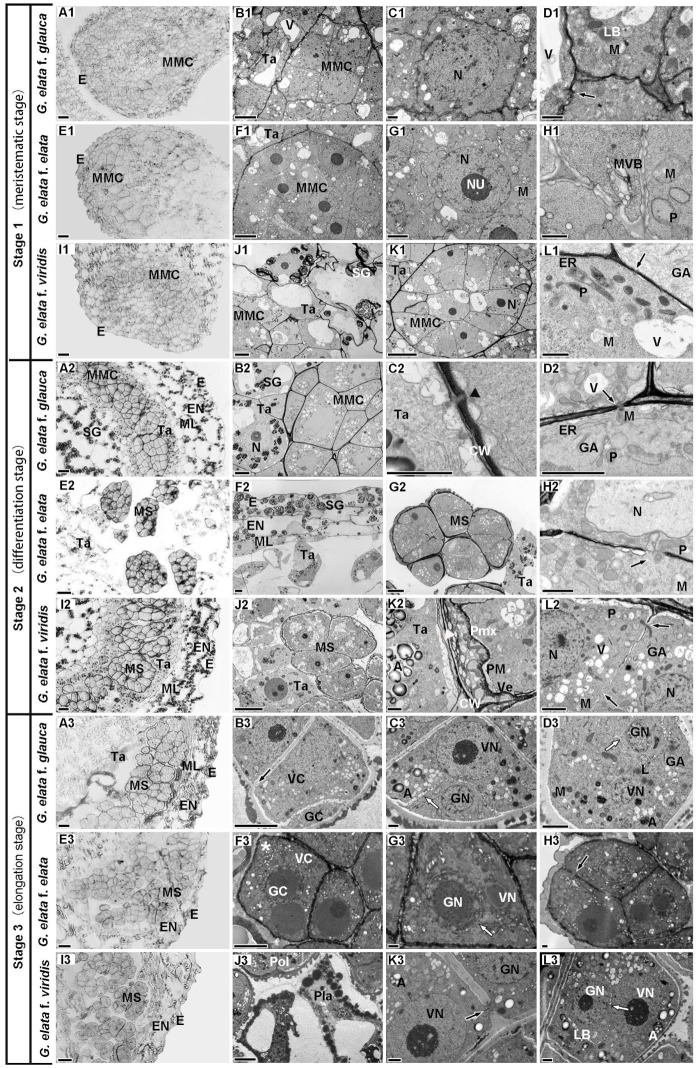
Microstructure of microsporogenesis and male gametophyte development in three *G. elata* forms at meristematic, differentiation and elongation stages. (**A1**–**D1**) *G. elata* f.* glauca*: microspore mother cells (MMC) at prophase I preparation stage. (**E1**–**H1**) *G. elata *f.* elata*: MMC at meiotic stage. (**I1**–**L1**) *G. elata *f. *viridis*: MMC at meiotic stage. (**A2**–**D2**) *G. elata* f. *glauca*: MMC at meiotic stage; (**E2**–**H2**) *G. elata* f. *elata*: microspores at mitotic stage. (**I2**–**L2**) *G. elata* f. *viridis*: microspore tetrad formation stage. (**A3**–**D3**) *G. elata* f. *glauca*: microspore mitosis and early pollen grain formation. (**E3**–**H3**) *G. elata* f. *elata*: microspore mitosis and mature pollen grains. (**I3**–**L3**) *G. elata* f. *viridis*: microspore mitosis and pollen grain formation. Annotations: black arrows = cytoplasmic channels; white arrows = callose wall of the generative cell; black triangles = pathways for material exchange between the tapetal cell wall and adjacent microspores; white triangles = tapetal cell wall specialized into a three-layered secretory barrier (electron-dense outer/inner edges, electron-lucent middle region); * = microspore cytoplasm protruding toward the anther locule. Abbreviations: A = amyloplast; CW = cell wall; E = epidermis; En = endothecium; ER = endoplasmic reticulum; GA = Golgi apparatus; GC = generative cell; GN = generative nucleus; LB = lipid body; M = mitochondrion; ML = middle layer; MMC = microspore mother cell; MS = microspore; MVB = multivesicular body; N = nucleus; PM = plasma membrane; Pmx = primexine; P = plastid; SG = starch grain; Ta = tapetum; V = vacuole; Ve = secretory vesicle; VC = vegetative cell; VN = vegetative nucleus. Scale bars: (**A1**,**E1**,**I1**,**A2**,**E2**,**I2**, **A3**,**E3**,**I3**) = 20 µm; (**B1**,**F1**,**J1**,**B2**,**F2**,**G2**,**J2**,**B3**,**F3**,**J3**) = 5 µm; (**C1**,**D1**,**G1**,**H1**,**K1**,**L1**,**C2**,**D2**,**H2**,**K2**,**L2**,**C3**,**D3**,**G3**,**H3**,**K3**,**L3**) = 1 µm.

At the meristematic stage (cytological phases 1–3, Table 2), microspore mother cells and anther walls initially differentiated: f. *glauca* had small nucleoli in microspore mother cells, active exocytosis of multivesicular bodies (Figure 4(B1–D1)), and 2–4 layers of anther walls (Figure 4(A1)); f. *elata* had obvious nucleoli, enriched mitochondria, and active exocytosis (Figure 4(F1–H1)), with 3–4 layers of anther walls and a small amount of starch granules in the epidermis and endothecium (Figure 4(E1)); f.* viridis* had 4–5 layers of anther walls (Figure 4(I1)), the epidermis and endothecium were rich in amyloplasts, the tapetum initiated vacuolization first, and nuclear states were asynchronous (Figure 4(J1–L1)).

**Figure 5:**
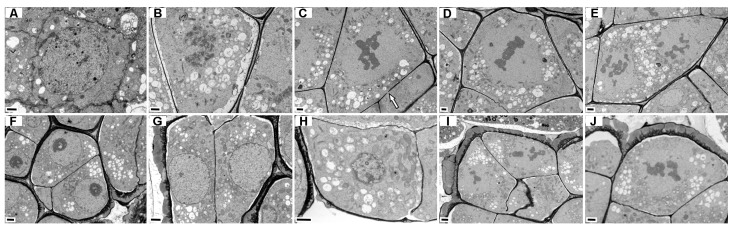
Meiosis of microsporocytes and mitosis of microspores in *G. elata* f.* glauca* and f.* elata*. (**A**–**E**) *G. elata* f.* glauca* meiosis: (**A**) Prophase I preparation; (**B**,**C**) Prophase I (arrow = cytoplasmic channel); (**D**) Metaphase I; (**E**) Prophase II. (**F**) *G. elata* f.* elata* microspore tetrad. (**G**–**J**) *G. elata* f*. elata* mitosis: (**G**,**H**) Prophase; (**I**,**J**) Metaphase. Scale bar = 1 μm.

At the differentiation stage, cytological stages differed significantly (Table 2): f.* glauca* was in stage 3 (meiosis), f. *elata* in stages 4–5 (microspore determination–mitosis), and f. *viridis* in stage 4 (microspore determination). Tapetum degradation initiated earliest in f.* elata*, while tapetal cells remained intact in f. *glauca* and f. *viridis* (Figure 4(A2,I2)). Continuous ultrastructures of microspore meiosis were observed in f.* glauca* (Figure 5(A–E)); microspores of f.* elata* entered the mitotic stage and developed into bicellular pollen (Figure 5(F–J)); f. *viridis* was still in the tetrad formation stage (Figure 4(J2–L2)).

At the elongation stage (cytological stages 5–6, Table 2), the anther wall structure simplified to 2–3 layers, and lignified spiral thickening occurred in the endothecium and outer middle layer (Figure 4(A3,E3,I3)); adjacent microspores in compound pollen were connected by cytoplasmic channels (Figure 4(B3,H3,I3)). Obvious membrane-wall gaps were found in microspores of f.* glauca* and f. *viridis*, with irregular generative cell contours and discontinuous bead-like callose walls (Figure 4(C3,D3,L3)). However, the generative nucleus of f.* glauca* was mostly condensed (Figure 4(C3)), while that of f. *viridis* had an obvious nucleolus (Figure 4(K3,L3)). The membrane-wall gap of microspores in f.* elata* almost disappeared, with cytoplasmic electron density significantly higher than the other two forms (Figure 4(F3–H3)); the generative cell was regularly round with a smooth and continuous callose wall (Figure 4(G3)). Quantitative analysis showed that the maximum endothecium thickness of f. *glauca* ranked first at the elongation stage, while that of f.* viridis* was the highest at the meristematic and differentiation stages, and f. *elata* was the lowest at all stages (Table 3); the cytoplasmic channel width of f.* glauca* was the narrowest but longest-lasting (from microspore mother cell meiosis to pre-pollen maturation, Figure 4(D2,B3)), while those of f. *elata* and f.* viridis* were wider but shorter-lasting (from tetrad to pre-pollen maturation, Figure 4(H2,L2,H3,K3)).

#### 2.4.2. Polar Assembly of Pollen Wall and Inter-Form Differences

**Figure 6:**
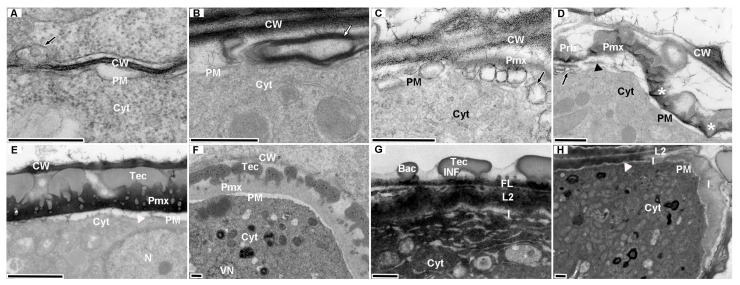
Ultrastructural dynamics of pollen wall development in three *G. elata* forms. (**A**,**B**) Callose wall stage: (**A**) *G. elata* f. *viridis*; (**B**) *G. elata* f. *glauca*. (**C**) Pmx formation stage (*G. elata* f. *viridis*; arrows: active exocytotic vesicles of PM). (**D**,**E**) Sporopollenin deposition stage: (**D**) *G. elata* f. *viridis* (tapetum-derived sporopollenin precursors deposited on Pmx, forming probaculae [Prb]); (**E**) *G. elata* f. *elata* (baculae [Bac] apices expand radially, forming discontinuous tectum [Tec]). (**F**) Callose wall degradation stage (*G. elata* f. *glauca*; callose wall enzymatically degraded/thinned). (**G**) Wall layer consolidation stage (*G. elata* f. *glauca*): Detail of the locular-side pollen wall with intact exine ornamentation, showing semitectate Tec, columellate infratectum (INF), continuous foot layer (FL), subjacent layer 2 (L2), and a thin intine (I). (**H**) New pollen tube wall formation stage (*G. elata* f. *elata*; intine separated from L2 layer, initiating new wall synthesis). Annotations: black arrows = exocytotic vesicles of microspore plasma membrane (PM); white arrows = exocytosed black annular substances from PM; * = sporopollenin embedded in the primexine; black triangles = electron-dense fibrillar materials abundant in the membrane-wall gap; white triangles = membrane-wall gap. Abbreviations: Bac = baculae; CW = cell wall; Cyt = cytoplasm; FL = foot layer; INF = infratectum; I = intine; L2 = layer 2; PM = plasma membrane; Prb = probaculae; Pmx = primexine; Tec = tectum; VN = vegetative nucleus. Scar bar = 500 nm.

Pollen wall assembly in the three forms all followed a six-stage program: callose wall formation (I) → primexine construction (II) → sporopollenin accretion (III) → callose wall degradation (IV) → wall layer consolidation (V) → new pollen tube wall formation (VI) (Figure 6), but significant asynchrony in process and structural morphology was observed at the same anther developmental stage (Table 2).

**Figure 7:**
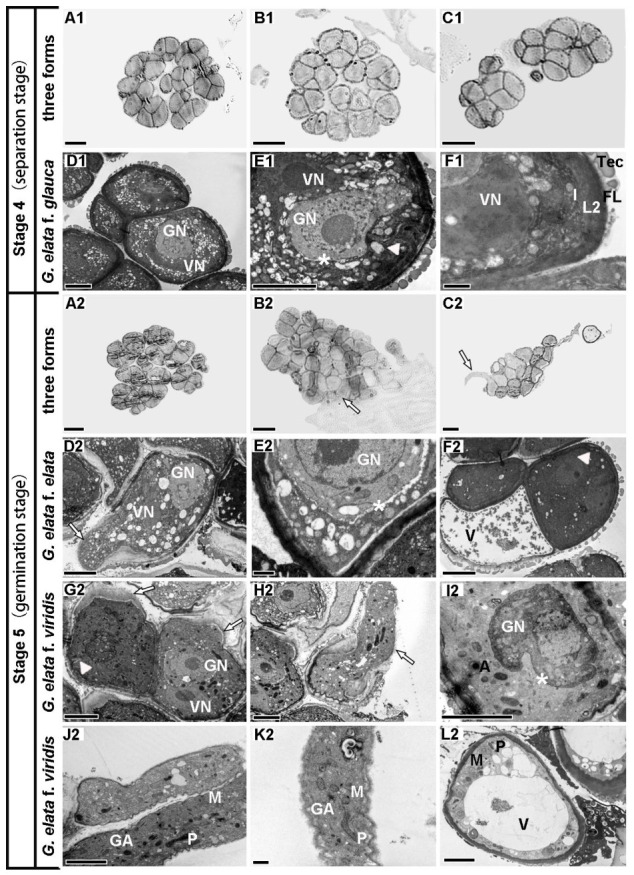
Microstructure of male gametophyte development and pollen germination in anthers of different *G. elata* forms at separation and germination stages. (**A1**–**C1**) Anthers at full anthesis: (**A1**) *G. elata* f. *glauca*, (**B1**) f. *elata*, (**C1**) f. *viridis*. (**D1**–**F1**) *G. elata* f. *glauca*: bicellular pollen with morphological change. (**A2**–**C2**) Anthers at 8 h after pollination: (**A2**) *G. elata* f. *glauca*, (**B2**) f. *elata*, (**C2**) f. *viridis*. (**D2**–**F2**) *G. elata* f. *elata*: (**D2**) pollen germination; (**E2**) callose wall of the generative cell still present; (**F2**) pollen cytoplasm degeneration and disappearance of organelles. (**G2**–**L2**) *G. elata* f. *viridis*: (**G2,H2,J2K2**) pollen grain germination; (**I2**) degradation of the generative cell callose wall; (**L2**) simplified pollen structure with degenerated nucleus. Annotations: white triangles = folded regions of the L2 layer; white arrows = pollen tube emergence; * = status of the generative cell callose wall. Abbreviations: A = amyloplast; FL = foot layer; GA = Golgi apparatus; GN = generative nucleus; I = intine; L2 = layer 2; M = mitochondrion; P = plastid; Tec = tectum; V = vacuole; VN = vegetative nucleus. Scale bars: (**A1**–**C1**,**A2**–**C2**) = 100 µm; (**D1**,**E1**,**D2**,**F2**–**J2**) = 5 µm; (**F1**,**E2**,**L2**) = 1 µm.

At the callose wall formation stage (I, Figure 6A,B), cytoplasmic channels (approximately 100 nm wide) formed in the tapetal cell wall, and the microspore plasma membrane released vesicles or secreted electron-dense annular substances, with no obvious inter-form differences (Table 2). At the primexine construction stage (II, Figure 6C), primexine formed between the plasma membrane and callose wall: f.* glauca* was in the I/II transition stage, f.* viridis* in the early-middle II/III stage, and f. *elata* had entered the late III stage (Table 2). At the sporopollenin accretion stage (III, Figure 6D,E), the tapetal cell wall specialized into a three-layer structure (electron-dense inner/outer layers, electron-transparent middle layer) (Figure 4(K2)); sporopollenin precursors were released into the anther locule and preferentially deposited on the locular-side primexine, forming primordial baculae, columellae, and a discontinuous tectum, with minimal deposition on the lateral walls, establishing the polar characteristic of “well-developed locular side, simplified lateral sides”. f.* elata* had densely arranged columellae, f. *viridis* had sparsely distributed columellae, and f.* glauca* was still in stage II (Table 2). At the callose wall degradation stage (IV, Figure 6F), the callose wall degraded and thinned: the primexine of f.* glauca* and f. *viridis* thickened significantly with an electron-transparent smooth structure (Figure 4(B3,K3)), while that of f. *elata* was a thin, electron-dense undulating structure (Figure 4(G3)). These ultrastructural differences are consistent with previously reported patterns in pollen wall development [21]. f.* glauca* and f. *viridis* entered the late IV stage, and f. *elata* the early-middle IV stage (Table 2). At the wall layer consolidation stage (V, Figure 6G), columellae on the locular side formed a locally continuous foot layer, constituting a complete pollen wall; mature non-germinated pollen of all three forms was in stage V (Table 2). At the new pollen tube wall formation stage (VI, Figure 6H), the anther endothecium separated from the L2 layer, with abnormal thickening in the early stage and recovery to normal thickness in the late stage (Figure 7(D2,G2)); only f.* elata* and f.* viridis* entered stage VI, and no germination was observed in f.* glauca* (Table 2).

The mature pollen wall of f. *glauca* exhibited two structural states: (1) a progressive decrease in electron density from the foot layer toward the intine, with the foot layer readily distinguishable from the L2 layer, but no clear boundary between the L2 layer and intine, and no interlayer gaps (Figure 7(F1)); (2) distinct stratification with an undulating L2 layer and obvious gaps between the L2 layer, foot layer, and intine (Figure 7(E1)). L2 layer folding was observed in mature and germinated pollen of all three forms, with form-specific folding positions: distributed at both proximal and distal poles in f. *elata* (Figure 7(D2,F2)), and only at the distal pole in f.* glauca* and f.* viridis* (Figure 7(E1,G2)), with folding direction inconsistent with pollen tube germination direction. Quantitative analysis at the elongation stage showed that the average thickness of the locular-side pollen wall of f.* glauca* (1.3 ± 0.2 μm) was significantly higher than that of f.* elata* (1.04 ± 0.23 μm) and f. *viridis* (1.03 ± 0.34 μm); the polar thickness ratio of f. *glauca* (4.8 ± 1.81) and f. *elata* (5.13 ± 1.49) was significantly higher than that of f.* viridis* (2.91 ± 0.52, *p* < 0.05, Table 4).

#### 2.4.3. Diversity in Male Gametophyte Maturation and Post-Pollination Response

At the elongation stage, the proportion of compound pollen reached 89–90% in all three forms, and remained at 81–87% at the separation stage (Figure 7(A1–C1)). Significant inter-form differences were observed in pollen grain size, callose wall status of generative cells, and nuclear characteristics at the separation stage (Table 5): f.* glauca* had the largest tetrad diameter (15.61 ± 2.18 μm), retained the callose wall of generative cells in mature pollen, and 80% of its pollen had nucleolus-free vegetative and generative nuclei (Figure 7(D1,E1)); f.* elata* had the smallest tetrad diameter (11.74 ± 0.66 μm), and the callose wall persisted until pollen tube germination (Figure 7(E2)); f.* viridis* showed intermediate traits, with the callose wall degrading after pollen tube formation (Figure 7(I2)).

At the germination stage, the pollen population of f.* elata* exhibited high developmental synchrony with an abortion rate of 22.22%. Germination initiated from the locular-side pollen wall, with 72.22% of pollen having crescent-shaped vegetative nuclei and 55.56% having round generative nuclei, all without nucleoli (Figure 7(D2–F2)). In contrast, the pollen population of f.* viridis* showed significant developmental asynchrony with an abortion rate of 33.33%; its pollen tubes were rich in organelles such as mitochondria and small vacuoles (Figure 7(J2,L2)), 77.78% of pollen had irregular vegetative nuclei and 71.43% had fusiform generative nuclei, with no nucleoli observed (Figure 7(G2–I2)); no germination structures were detected in f. *glauca* (Figure 7(A2)).

### 2.5. Ultrastructure of Megasporogenesis and Female Gametophyte Development

**Figure A3:**
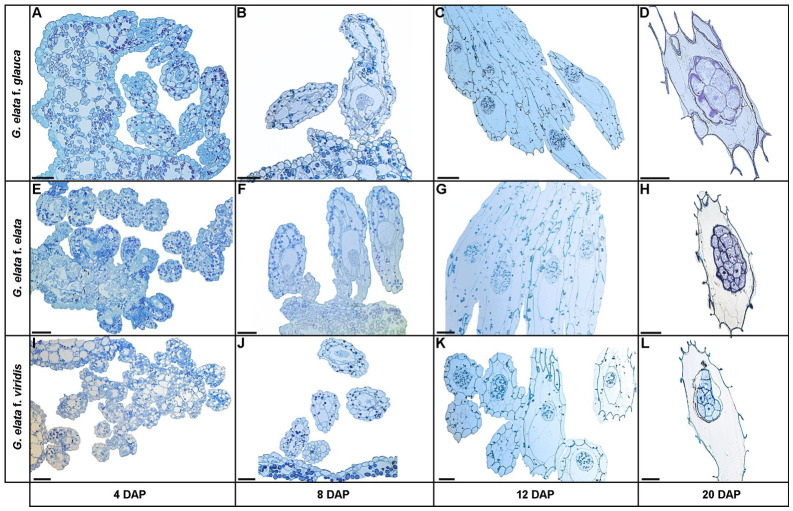
Light microscopic observations of ovary semi-thin sections in three *G. elata* forms at 4, 8, 12, and 20 DAP. (**A**–**D**) *G. elata* f. *glauca*. (**E**–**H**) *G. elata *f.* elata*. (**I**–**L**) *G. elata *f. *viridis*. Scale bars: (**A**,**B**,**D**–**L**) = 50 µm; (**C**) = 100 µm.

Ovule development was highly asynchronous within the same ovary of the three forms, with coexisting stages including archesporial cells, megaspore mother cells, dyads, functional megaspores, and mature embryo sacs (Figure A3). Fertilization-related signs were observed in all three forms at 4 DAP (see Section 2.6), and ovules at the triad and mature embryo sac stages were still found in the ovary of f. *elata* at 8 DAP.

#### 2.5.1. Megasporogenesis Stage

**Figure 8:**
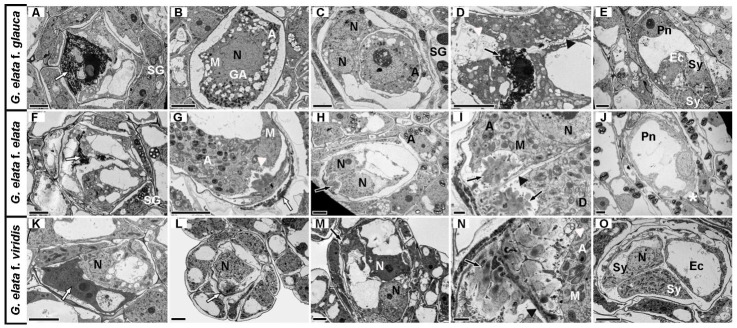
Comparative stages of megasporogenesis and megagametogenesis in three *G. elata* forms. (**A**–**E**) *G. elata* f. *glauca*: (**A**) triad of megaspores; (**B**) functional megaspore; (**C**) tetranucleate embryo sac formation; (**D**) tetranucleate embryo sac degeneration; (**E**) early mature embryo sac. (**F**–**J**) *G. elata* f. *elata*: (**F**) triad of megaspores; (**G**) functional megaspore; (**H**,**I**) tetranucleate embryo sac degeneration; (**J**) mature embryo sac. (**K**–**O**) *G. elata* f. *viridis*: (**K**) triad of megaspores; (**L**) functional megaspore; (**M**,**N**) tetranucleate embryo sac degeneration; (**O**) early mature embryo sac. Annotations: white arrows = degenerating micropylar megaspores; black arrows = degenerated nucleocytoplasmic remnants in the embryo sac; white triangles = abundant vesicles released toward the micropyle from functional compartments (**D**,**N**) or functional megaspore (**G**); black triangles = trace-like structures fading toward the chalazal end; * = nucellar cell. Abbreviations: A = amyloplast; Ec = egg cell; GA = Golgi apparatus; M = mitochondrion; N = nucleus; Pn = polar nucleus; SG = starch grain; Sy = synergid. Scale bars: (**A**–**H**,**J**–**M**,**O**) = 5 µm; (**I**,**N**) = 1 µm.

At 4 DAP, the megaspore mother cell formed three haploid megaspores after meiosis; the two non-functional megaspores near the micropyle underwent programmed degradation sequentially (nuclear membrane disintegration, cell structure simplification, increased electron density) (Figure 8A,F,G,K,L), and only one chalazal megaspore differentiated into a functional megaspore (large vacuole formation, organelle enrichment, active exocytosis) (Figure 8B,G,N). Significant inter-form differences were found in the organelle composition of functional megaspores: f. *glauca* contained 1–14 early amyloplasts, 6–26 mitochondria, 1–12 Golgi apparatuses, and “1 large + multiple small” vacuoles; the proportion of mature amyloplasts in nucellar epidermal cells was 66.67–100% (Figure 8B). f. *elata* had 3–36 amyloplasts, 12–30 mitochondria, and a single large vacuole; the proportion of mature amyloplasts in nucellar epidermal cells was 60–66.67% (Figure 8G). f. *viridis* contained 2–18 early amyloplasts, 7–59 mitochondria, few Golgi apparatuses, and weak vacuole development; only early amyloplasts were found in nucellar epidermal cells (Figure 8L).

#### 2.5.2. Female Gametophyte Development Stage

At the four-nucleate embryo sac stage (4 DAP), all three forms showed characteristics such as binuclear coexistence, nuclear state differentiation, and degeneration; dense vesicle clusters were distributed near the interface between non-degenerated compartments and the micropylar degenerated region (Figure 8D,I,N). The four-nucleate embryo sac of f.* glauca* was in an undivided binuclear coexistence state (Figure 8C); the cytoplasm of the functional compartment was rich in amyloplasts, mitochondria, Golgi apparatuses, and small vacuoles, and 71.43–85.71% of nucellar epidermal cells contained early amyloplasts (Figure 8C,D). f. *elata* formed a binuclear compartment (one nucleus with a nucleolus, one without) (Figure 8H); the active compartment contained both amyloplasts and proplastids, and 37.5% of nucellar epidermal cells had mature amyloplasts. f. *viridis* had a binuclear compartment with obvious binucleoli (Figure 8M); the cytoplasm was rich in proplastids and mitochondria with small diameters, and only 18.18–44.44% of nucellar epidermal cells contained early amyloplasts.

From the third mitosis to the mature embryo sac stage (4 DAP), f.* glauca* had formed an incompletely cellularized four-compartment structure (Figure 8E), and initial callose wall deposition was observed between some compartments of f.* viridis* (Figure 8O). Mature embryo sacs were observed in f.* elata* at 4 DAP, and their ultrastructures were clearly observed at 8 DAP (Figure 8J): the central cell was dominated by a large vacuole, with the nucleus and cytoplasm squeezed to the edge; long obtriangular nucellar cells were present outside the embryo sac. Notably, between the functional compartment and the micropylar degenerated compartment of the three forms, in addition to vesicle clusters, there were fluid-like degradation product accumulation zones and trace-like structures fading toward the chalazal end, consistent with typical intermediate characteristics of PCD degradation (Figure 8(D,I,N)) [27]. It is tentatively speculated that these structures may be involved in the transport and spatial reorganization of degraded substances, although direct evidence (e.g., vesicle tracing) is lacking.

### 2.6. Ultrastructure of Fertilization and Embryogenesis

#### 2.6.1. Fertilization and Zygote Formation

**Figure 9:**
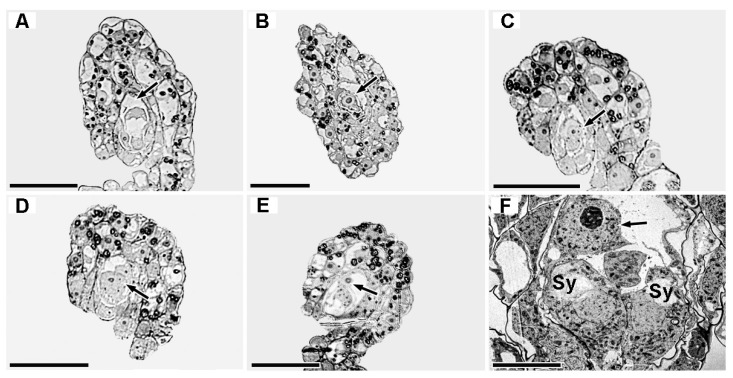
Microstructure of fertilization and zygote formation in three *G. elata* forms at 4 DAP. (**A**) *G. elata *f.* glauca*: two sperm nuclei entering the mature embryo sac (arrow = contact between sperm and polar nuclei). (**B**) *G. elata *f.* glauca*: zygote (transverse section, arrow = zygote) and primary endosperm nucleus. (**C**) *G. elata *f*. elata*: fusion of sperm and polar nuclei (arrow). (**D**) *G. elata *f.* elata*: sperm nucleus entering the egg cell (arrow). (**E**) *G. elata *f.* elata*: zygote (longitudinal section, arrow = nucellar epidermis cell nucleus). (**F**) *G. elata *f. *viridis*: fusion of sperm and polar nuclei (arrow). Abbreviations: Sy = synergid. Scale bars: (**A**–**E**) = 50 µm; (**F**) = 10 µm.

At 4 DAP, ovules of f. *glauca* showed coexisting multiple stages, with sperm nucleus-polar nucleus contact, zygotes, and primary endosperm nuclei visible (Figure 9A,B); f. *elata* developed faster, with sperm nucleus entry into the egg cell, sperm nucleus-polar nucleus fusion, and nucellar epidermal cell wall disintegration surrounding the zygote observed simultaneously in the same ovary (Figure 9C–E); f. *viridis* developed slowly, with only polar nucleus–sperm nucleus fusion observed and no zygote or proembryo structures found (Figure 9F).

#### 2.6.2. Early Proembryo Development and Integument Differentiation

**Figure 10:**
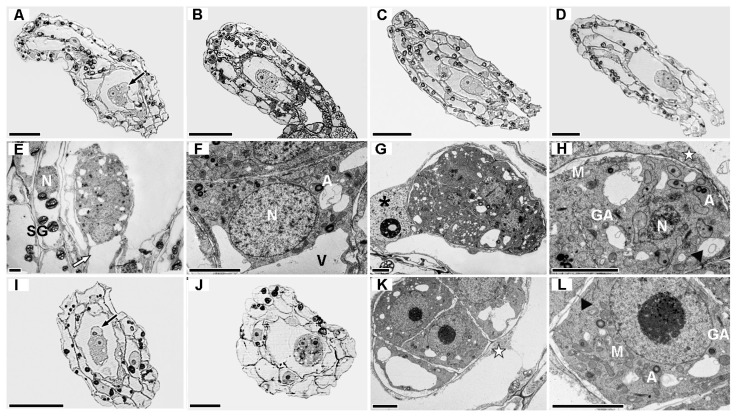
Microstructure of embryos in three *G. elata* forms at 8 DAP. (**A**) *G. elata* f. *glauca*: basal cell undergoing the first transverse division. (**B**–**E**) *G. elata *f.* elata*: basal cell with transverse division and apical cell with longitudinal division. (**F**) *G. elata *f. *elata*: suspensor cell. (**G**) *G. elata *f.* elata*: embryo surrounded by integument cytoplasm. (**H**) *G. elata *f.* elata*: callose wall formation in the apical cell. (**I**–**K**) *G. elata* f.* viridis*: embryos with different cell numbers. (**L**) *G. elata* f. *viridis*: embryonic cells. Annotations: black arrows = endosperm nucleus; white arrows = suspensor cell; * = naked integument nucleus; black triangles = plasmodesmata between embryonic cells; white stars = degraded integument cytoplasm. Abbreviations: A = amyloplast; GA = Golgi apparatus; M = mitochondrion; N = nucleus; SG = starch grain; V = vacuole. Scale bars: (**A**–**D**,**I**,**J**) = 50 µm; (**E**–**H**,**K**,**L**) = 5 µm.

At 8 DAP, f.* glauca* entered the early proembryo stage with 2-celled proembryos visible; the primary endosperm nucleus had a clear nucleolus, the integument closed, and the ovule separated from the placenta (Figure 10A). f.* elata* showed significant developmental asynchrony, with 2–6-celled proembryos found in the same ovary; suspensor cells contained large vacuoles, and the integument was not closed (Figure 10B–F); the embryo was surrounded by integument cytoplasm with naked nuclei (Figure 10G,H). f.* viridis* entered the proembryo stage with the integument closed, 3-celled proembryos and primary endosperm nuclei visible (Figure 10I); the embryo was surrounded by degraded integument cytoplasm (Figure 10K,L).

#### 2.6.3. Proembryo Proliferation and Embryo Envelope Formation

**Figure 11:**
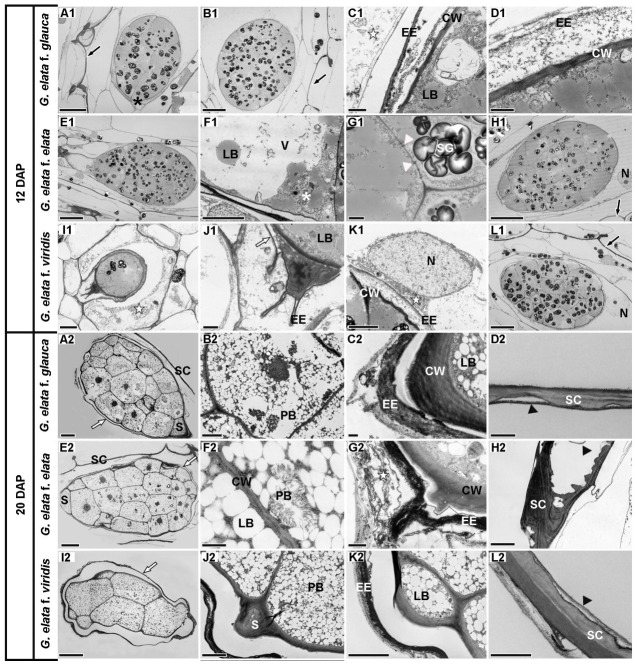
Ultrastructure of embryos in three *G. elata* forms at 12 and 20 DAP. (**A1**–**L1**) 12 DAP; (**A2**–**L2**) 20 DAP. Annotations: black arrows = secondary thickening of the inner tangential and radial walls; black asterisks = long inverted triangular nucellar cells immediately adjacent to the basal part embryo in *G. elata* f. *glauca*; white stars = degraded integument cytoplasm; white asterisks = condensed chromatin dispersed as electron-dense threads of varying lengths during suspensor cell degeneration; white triangles = plasmodesmata between embryonic cells in *G. elata* f.* elata*; white arrows = embryo envelope; black triangles = membrane structure on the seed coat surface of all three forms. Abbreviations: CW = cell wall; EE = embryo envelope; LB = lipid body; N = nucleus; PB = protein body; S = stalk cell; SC = seed coat; SG = starch grain; V = vacuole. Scale bars: (**A1**,**B1**,**E1**,**H1**,**L1**,**A2**,**E2**,**I2**) = 20 µm; (**F1**,**I1**,**K1**,**B2**,**H2**,**J2**,**K2**) = 5 µm; (**C1**,**D1**,**G1**,**J1**,**C2**,**D2**,**F2**,**G2**,**L2**) = 1 µm.

At 12 DAP, all three forms entered the rapid proembryo proliferation stage, with embryo envelope formation and initial seed coat differentiation occurring synchronously. The number of embryonic cells was 4–20 in f.* glauca*, 2–22 in f.* elata* (including intact 18–20-celled proembryos), and 8–14 in f. *viridis* (including unicellular proembryos); the embryonic cytoplasm was dense and rich in starch granules (Figure 11(A1,E1,L1)). A rudimentary embryo envelope composed of black flocculent material formed around the embryo (0.14–0.52 μm in f. *glauca*, 0.12–2 μm in f.* elata*, 0.15–1.38 μm in f. *viridis*) (Figure 11(C1)), and the embryo envelope was surrounded by integument cytoplasm (except for unicellular proembryos) (Figure 11(H1,K1)). The outer tangential wall of the outer embryonic cells thickened significantly, while the inner cell wall was thin, with the ratio of outer to inner wall thickness being 1:5–1:10 in f.* glauca*, 1:4–1:6 in f. *elata*, and 1:8–1:25 in f.* viridis*. A large number of medium-gray spherical lipid bodies and mature amyloplasts were distributed in the cytoplasm (Figure 11(G1)). Notably, the suspensor of the 18–20-celled proembryo of f.* elata* began to degenerate, with the nucleus disintegrating into electron-dense threads, indicating chromatin condensation and nuclear fragmentation (Figure 11(F1)); unicellular proembryos were still found in f. *viridis*, with irregular degenerated suspensor remnants at the base surrounded by the embryo envelope, and nucellar epidermal cells initiating degradation (Figure 11(I1,J1)). The integument cell walls of the three forms all showed the polar characteristic of secondary thickening of the inner tangential and radial walls and no thickening of the outer tangential wall, with simplified cell structures (Figure 11(B1,H1,L1)).

#### 2.6.4. Seed Maturation

At 20 DAP, seeds were basically mature, but significant form-specific characteristics were observed in the number of embryonic cells, storage substances, and seed coat structure (Table A2): the number of embryonic cells followed the order f.* elata* (23–31) > f.* glauca* (21) > f.* viridis* (7–10), with all stalk cells being obtriangular (Figure 11(A2,E2,I2)); Figure A3). A dark, electron-dense embryo envelope with a clear edge surrounded the embryo (0.37–1.88 μm in f.* glauca*, 0.18–1.86 μm in f.* viridis*, 0.19–0.66 μm in f. *elata*) (Figure 11(A2,E2,I2)); a small amount of incompletely degraded integument cytoplasm remained outside the embryo envelope, with the residual amount in f. *viridis* significantly less than that in f. *glauca* and f. *elata*. Suspensor cells degenerated completely, and the cell wall at the junction of stalk cells and degenerated suspensors thickened significantly (Figure 11(C2,G2,K2)).

Storage substances in embryonic cells were mainly observed as white spherical lipid bodies and protein bodies; some seeds of f. *elata* had medium-gray lipid bodies and additionally retained a small amount of mature amyloplasts (Figure 11(E2,F2)). Protein body morphology was form-specific: spherical with dark flocculents in f. *glauca* (long axis 0.95–15.81 μm, Figure 11(B2)); spherical with acicular phytin crystals in f. *elata* (diameter 0.54–2.99 μm, Figure 11(F2)); and spherical in f.* viridis* (diameter 0.55–6.43 μm, Figure 11(J2)). The polar thickening mode of the seed coat was conservative (consisting of a basal layer and protrusions), but the thickening pattern of the inner tangential wall (slightly undulating in f. *glauca* and f. *viridis*, triangular-serrate in f. *elata*) (Figure 11(D2,H2,L2)) and the length of radial wall protrusions (f.* viridis* > f.* glauca* > f.* elata*) (Figure A3(D,H,L)) were form-specific, and a membrane structure was present on the seed coat surface of all three forms (Figure 11(D2,H2,L2)).

In summary, fertilization and embryogenesis in the three forms followed a conservative program, but obvious form-specific characteristics were found in developmental rhythm and structural traits (Table A3): f.* elata* developed fastest, forming zygotes and initiating nucellar epidermal cell degradation at 4 DAP, with 23–31 embryonic cells at 20 DAP; f.* glauca* was intermediate, at the zygote stage at 4 DAP with 21 embryonic cells at 20 DAP; f. *viridis* developed slowest, at the sperm-egg fusion stage at 4 DAP, with unicellular proembryos still present at 12 DAP and only 7–10 embryonic cells at 20 DAP. Seed coat differentiation all followed the program of outer tangential wall degradation and secondary thickening of inner tangential and radial walls, but the thickening pattern of the inner tangential wall and the length of radial wall protrusions were form-specific.

## 3. Discussion

### 3.1. Polar Differentiation and Simplified Characteristics of Pollen Wall Development

Referring to the classical framework of pollen wall development established by Hu [28] based on *Helleborus* sp., we systematically traced the complete process of pollen wall development in *G. elata* from callose wall formation to pollen tube emergence, and for the first time defined a six-phase developmental program. This dynamic model integrates the coordinated action of the sporophyte (tapetum) and gametophyte (microspore) throughout pollen wall construction, filling a gap left by previous studies that focused mainly on mature structures [29]. Compared with Purgina et al.’s [21] observations on mature pollen wall structures of five Epidendroideae species, our time-course analysis provides a more complete cytological basis for understanding pollen wall simplification in orchids.

The highly polar distribution of sporopollenin in the pollen wall, characterized by the pattern “well-developed on the locular side, reduced on the lateral sides”, reflects spatial heterogeneity in sporopollenin synthesis and deposition. This polarity likely involves two key factors: (1) the narrow lateral space restricts the diffusion and transport of tapetum-derived sporopollenin precursors [30]; and (2) the primexine on the lateral sides has lower affinity for sporopollenin deposition than that on the locular side. This template function difference may stem from distinct properties established during primexine formation—regulated by plasma membrane-associated proteins, the primexine can form regions of varying electron density via physical phase separation [31,32]. Sporopollenin synthesis relies on the oxidative polymerization of fatty acids and phenolic compounds [22], with the tapetum as the main site of precursor synthesis [33]. Recent studies show that microspores actively participate in sporopollenin phenolic unit polymerization via SCULP1 [34], and bidirectional signal communication exists between the tapetum and microspores [35], forming a synergistic network of “tapetal synthesis—directional transport—microspore autoregulation”. On the locular side of *G. elata*, this network ensures sufficient precursor supply and efficient deposition. In contrast, lateral sides suffer from restricted precursor transport and insufficient microspore autoregulation activation, leading to reduced sporopollenin deposition and the “one-side developed, one-side simplified” structural pattern. Sporopollenin synthesis-related genes (e.g., CYP703A, ACOS5 [36,37,38]) are likely molecular drivers of these inter-form structural differences.

This structural pattern differs from the uniform sporopollenin deposition in soft pollinia species (e.g., *Cephalanthera longifolia*) [21] and the peripheral concentration gradient in hard pollinia species (e.g., *Bulbophyllum retusiusculum*) [21,39,40]. It likely represents a transitional evolutionary feature of the farinaceous pollinia of *G. elata* [41,42]. Given the basal phylogenetic position of Gastrodieae within Epidendroideae [43], we propose this pattern as an intermediate state in the evolution from “uniform distribution” to “peripheral concentration”. It reflects an adaptive trade-off in mycoheterotrophic orchids: directional sporopollenin deposition (prioritizing locular-side function) reduces energy consumption while maintaining basic pollen dispersal and fertilization capabilities.

Inter-form differences in L2 layer electron density (transparent in f.* glauca* and f. *viridis*, electron-dense in f.* elata*) align with the “polysaccharide-sporopollenin composite model” [21,44]. Based on these ultrastructural characteristics, High electron density in f.* elata* may reflect earlier, denser sporopollenin granule deposition, while the transparent state in f.* glauca* and f.* viridis* suggests a higher polysaccharide matrix proportion—consistent with their developmental rhythms (fastest in f. *elata*). We speculate *G. elata* accelerates sporopollenin synthesis and deposition via enhanced tapetum-microspore metabolic communication, a process potentially regulated by core sporopollenin biosynthesis genes [36,37,38]. Variations in cytoplasmic channel width and duration further support this mechanism: wider channels enhance transport efficiency of precursors like acetyl-CoA [36,45], providing sufficient substrate for sporopollenin polymerization [34] and promoting pollen wall maturation; narrow channels restrict precursor transport [34,46], impairing male gametophyte development.

### 3.2. Inter-Form Specificity in Male Gametophyte Maturation and Germination

The “functional condensed state” of mature pollen nuclei (nuclear envelope disintegrated, no nucleolus, chromatin condensed) is a conserved feature of *G. elata* male gametophytes, consistent with bicellular pollen specialization in other Epidendroideae species [21,29]. Liang [19] reported “prominent nucleoli” in *G. elata* pollen tubes, differing from our observations in mature pollen. Combined with the dynamic “mature condensation—germination activation” changes in generative nuclei reported for *Lilium longiflorum*, *Brassica napus*, and *Nicotiana tabacum* [47,48,49], we suggest that generative nuclei of *G. elata* also transition from a “transport-dormant” to a “fertilization-activated” state. These two observations represent successive stages in the complete developmental trajectory of the generative nucleus.

Core inter-form differences lie in the coordination between pollen germination and generative cell callose wall dynamics: f.* elata* exhibits high germination synchrony and low abortion rate, with callose walls persisting until pollen tube germination; f. *viridis* shows pronounced developmental asynchrony and higher abortion rate, with callose walls degrading after typical pollen tube formation; f. *glauca* displays no germination structures and retains callose walls in mature pollen. Although all materials were grown under uniform controlled conditions in Beijing, these differences likely reflect intrinsic traits adapted to natural vertical distributions: f.* elata* (low elevation) may have evolved a “rapid germination” strategy, while f.* glauca* (high elevation) delays development via “no germination + callose wall retention” to adapt to low temperatures [15]. Notably, Zhou et al. [10] and Liang [18,19] reported generative cell callose wall degradation before pollen maturation in Yunnan f. *glauca*, contrasting with retention observed in Hubei f.* glauca* here. This discrepancy may reflect intraspecific natural variation [6], similar to population-level reproductive strategy variation reported in *Ranunculus kuepferi* [50]. Studies of arabidopsis show callose wall degradation is regulated by cell wall integrity signaling [51], suggesting a similar mechanism may operate in *G. elata*. The conserved distal pollen germination sitealigns with the distal monocolpate aperture common in many Epidendroideae species [52]. Inter-form differences in L2 folding location (f.* elata*: proximal + distal folds; f. *glauca* and f. *viridis*: distal-only folds) are unrelated to germination direction. This suggests precise aperture positioning is independently regulated by factors such as meiotic cytokinesis pattern, callose deposition, and specific proteins [53], with no direct link to L2 mechanical properties.

### 3.3. Nuclear Clearance and Directional Transport of Remnants in Female Gametophyte Development

Based on ultrastructural observations, we tentatively propose a two-step model for *G. elata* female gametophyte development: “chalazal degradation—micropylar localization of remnants”. Light microscopy studies confirmed two chalazal nuclei degenerate before embryo sac maturation [18,19,20], but their remnant fate remained unclear. We observed vesicle clusters, fluid-like degradation product aggregates, and trace-like structures between functional compartments and the degenerating micropylar region, suggesting that these structures might mediate directional transport of degenerating nuclear remnants. However, this interpretation is indirect and requires validation by direct approaches such as immunocytochemistry using autophagy markers (e.g., ATG8) or fluorescent vesicle tracing. This process aligns with “vesicle-mediated packaging and transport of degradation products” during PCD in *Hordeum vulgare* aleurone cells [27]. Vesicle transport is conserved in plant reproductive development: in Arabidopsis, loss of COPII vesicle component Sar1 function inhibits functional megaspore mitosis and disrupts embryo sac development [54]; in *Capsella bursa-pastoris*, autophagic vesicles derived from nuclear envelope budding during female meiosis engulf and degrade degenerating organelles [55]. These cross-species findings support vesicle transport’s core role in directional processing of degenerating materials in the female gametophyte. In summary, *G. elata*’s simplified embryo sac couples with an efficient nuclear clearance mechanism. Concentrating degenerating remnants at the micropylar end frees metabolic space for functional chalazal nuclei (central cell nucleus, egg nucleus) while preventing degradation product interference with the egg apparatus—representing an adaptive trade-off between structural simplification and functional optimization in mycoheterotrophic plants. Future studies combining vesicle tracing and live-cell imaging could further validate this model. This represents a hypothetical adaptive trade-off that needs experimental testing.

### 3.4. Embryonic Development Rhythm and Resource Allocation Strategy

Embryo development rates differed markedly among the three forms and correlated closely with macro-phenotypic traits. f.* elata* developed fastest, with the highest embryo cell count and seed viability—consistent with the general principle that orchid seed viability correlates positively with embryo cell number [25]. Its compact “early flowering—early maturation” pattern, combined with strong vegetative growth, represents a “high-investment” strategy. The largest embryo volume confirms embryo volume as a valid seed quality indicator [26]. f. *glauca* and f.* viridis* had similar phenology but divergent embryo development: f.* glauca* had embryo cell numbers comparable to f.* elata* but the lowest viability, while f.* viridis* had the fewest embryo cells but intermediate viability. The TTC assay reflects potential seed metabolic activity, not actual germination ability [56,57]; the lack of a strict cell number–viability correlation suggests embryo development quality depends on both cell quantity and metabolic activity. Low f.* glauca* viability despite high cell numbers may relate to low-temperature stress at its high-altitude origin [15]; f. *viridis* may compensate for fewer cells with high-quality storage materials accumulated over a longer development period, forming a “slow development—high quality” strategy [58]. From an ecological perspective, inter-form development rate differences likely correspond to elevational distributions: f. *elata* (low elevation) “fast pace—high reserves”; f. *glauca* (high elevation) “multi-cell—low activity”; and f. *viridis* (mid-elevation) “slow pace—stable quality”. This is likely a result of long-term adaptation to different elevational environments.

### 3.5. PCD Characteristics of Suspensor and Maternal Tissues

Suspensor cell degeneration varied among forms. Li et al. [20] reported *G. elata*’s unicellular suspensor degenerates as the proembryo proliferates. We observed suspensor nuclei disintegrating into electron-dense threads—similar to nuclear changes during *Vicia faba* suspensor PCD [59], confirming this as a PCD process. By 20 DAP, the suspensor had fully degenerated, with a thick cell wall forming at the junction between stalk cells and degenerated suspensor remnants. Fungal hyphae aggregate around suspensor remnants and invade stalk cells, with papillae-like thickenings at invasion sites [12], suggesting this thickened cell wall may relate to specific localization of fungal invasion.

This study is the first to report PCD of nucellar epidermal and integument cells at the ultrastructural level, with inter-form timing differences: f.* elata* nucellar epidermal cells began degenerating at 4 DAP (releasing naked nuclei), while this process was delayed in f.* glauca* and f. *viridis*—correlating with fertilization progress (zygote stage in f. *elata* and f.* glauca*, sperm-egg fusion stage in f.* viridis*). This suggests nucellar epidermal cell PCD timing may reflect female gametophyte sensitivity to fertilization signals. Subsequently, integument cells began degenerating at 8–12 DAP; by 20 DAP, only the outermost integument cells remained, with their cell walls undergoing polar thickening to form the seed coat. This “nucellar epidermis first, integument later” temporal sequence aligns with the conserved angiosperm seed development pattern [23,24]: nucellar tissue PCD creates space for embryo sac expansion [23], integument cells degenerate progressively, and their breakdown products are remobilized to the developing embryo [24,60], with remaining cells forming the seed coat. Early nucellar epidermal cell PCD onset in f.* elata* matches its rapid development strategy, while delayed responses in f. *glauca* and f. *viridis* align with their slower rhythms—indicating coordinated regulation between maternal tissue degradation and embryo development.

### 3.6. Differentiation of Storage Substances and Structures in Mature Seeds

Mature seed storage materials varied significantly among forms. In plant seeds, protein bodies are the main protein storage organelles, often containing phytin-rich globoids. Under TEM, globoids appear as needle-like radiating structures, dark black flocculent material, or punctate clumps depending on section plane [61,62,63]. In f.* glauca* and f.* viridis*, protein bodies were mostly spherical with dark black flocculent material or punctate clumps; in f.* elata*, protein bodies coexisted with needle-like phytin crystals, and a few mature amyloplasts were retained. This lipid body-protein body co-localization is consistent with observations in *Amaranthus hypochondriacus* [64]. Zhou et al.’s [10] histochemical studies showed *G. elata* embryos have abundant oil droplets at 8 DAP, peak polysaccharide content at 16 DAP, and no detectable storage proteins. This does not contradict the abundant globoids observed here—globoids primarily store mineral elements [61], not proteins. Together, these findings suggest *G. elata* seeds have a coordinated storage system: lipid bodies provide energy, globoids supply mineral nutrients, and amyloplasts in f.* elata* offer an additional carbon source. During germination, protein bodies gradually degrade and vacuolate [63], while phytin is hydrolyzed by phosphatase to release inorganic phosphorus, potassium, magnesium, and calcium for seedling growth [61,65]. Retention of needle-like phytin crystals and amyloplasts in f. *elata* may provide more abundant carbon and mineral supplies for symbiotic germination, consistent with its highest seed viability. Note that our protein body identification relies primarily on morphological features and literature comparisons (morphological inference). Future studies using X-ray energy-dispersive analysis could verify globoid elemental composition [61,62].

This study is the first to report inter-form differentiation of embryo envelope and seed coat structure at the ultrastructural level. A dark black embryo envelope surrounded mature embryos, with thickness varying among forms—its formation may relate to cuticular material deposition [20]. As an embryo outer barrier, envelope thickness differences may affect initial fungal contact efficiency [12]. Seed coat development followed the common “outer tangential wall degeneration, inner tangential and radial wall secondary thickening” pattern [66], but thickening morphology was form-specific: inner tangential walls were slightly undulate in f.* glauca* and f.* viridis*, triangular-serrate in f. *elata*; radial wall protrusion length followed f.* viridis* > f.* glauca* > f.* elata*. These two wall thickening types might have distinct functions: long, sawtooth-like radial protrusions could alter seed aerodynamic properties to facilitate wind dispersal [25,66], with longer protrusions in f*. viridis* and f.* glauca* possibly being better adapted to high-altitude wind dispersal needs. Wavy/serrate inner tangential wall thickening might relate to seed coat mechanical strength and water permeability [66,67]. Combined with studies showing seed coat lignin restricts germination and mycorrhizal fungi promote germination by supplying nitrogen and auxin [68,69], we tentatively suggest that f.* elata*’s triangular-serrate inner tangential wall could facilitate water uptake and fungal invasion—consistent with its high seed viability. However, these functional interpretations are speculative and require direct experimental validation, such as wind tunnel assays to test dispersal efficiency and fungal inoculation experiments to assess germination enhancement. The function of the seed coat surface membrane layer requires further investigation.

### 3.7. Limitations and Future Perspectives

This study systematically characterized the cytological features of reproductive development in three *G. elata* forms, but several limitations remain. First, the molecular mechanisms underlying inter-form differentiation are unclear. Future studies should combine transcriptomics and proteomics to identify key genes regulating pollen wall polarity, programmed cell death, and embryo development rate. In particular, the proposed two-step nuclear clearance model requires direct experimental validation (see Section 3.3). Second, adaptive significance of observed reproductive traits is inferred from habitat distribution–phenotype correlations, lacking direct evidence from reciprocal transplant experiments and environmental factor manipulation. Furthermore, the source tubers were collected from geographically distinct regions and cultivated under common conditions for only one generation; thus, the observed inter-form differences may partially reflect maternal effects or environmental carry-over effects. Multi-generation common garden experiments would be necessary to confirm the genetic basis of the observed reproductive traits. Third, the relationship between seed coat structure (e.g., radial wall protrusions) and seed germination—particularly interactions with symbiotic fungi—has not been explored. Further studies focusing on seed coat traits in fungal recognition and colonization could inform artificial germination technique optimization. Additionally, purely technical sample preparation limitations led to missing information for critical stages: no valid ultra-thin sections for f.* elata* and f.* viridis* separation stages (preventing complete mature pollen wall thickness comparison); due to the prolonged duration of the elongation stage, the brief window of microspore mitosis in f.* glauca* was not captured in our sections (requiring additional elongation stage sampling), and late 4 DAP sampling (missing fertilization and zygote formation ultrastructural details, necessitating earlier time points like 2 DAP in future studies). Addressing these gaps will deepen the understanding of *G. elata* reproductive development regulatory mechanisms.

## 4. Materials and Methods

### 4.1. Plant Materials and Cultivation

**Figure A4:**
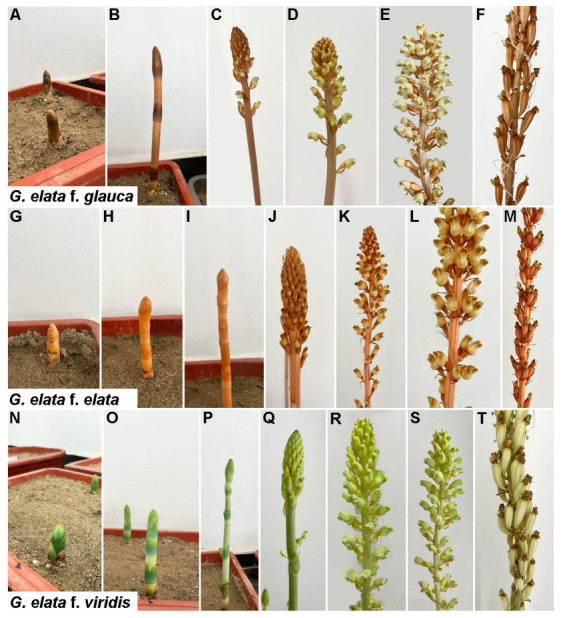
Morphological development from flower bud initiation to fruiting in three *G. elata* forms. (**A**–**F**) *G. elata *f. *glauca*. (**G**–**M**) *G. elata* f.* elata*. (**N**–**T**) *G. elata* f.* viridis*.

*G. elata *f.* glauca*, f. *elata*, and f. *viridis* were collected from Wufeng County (Yichang, China), Lueyang County (Hanzhong, China), and the Wumeng Mountain area (Yunnan-Guizhou border), respectively. Fifteen arrow tubers with intact terminal buds, uniform size, and no diseases/insect pests were selected for each form, sand-stored at 4 °C for 3 months, and planted in flowerpots under natural temperature and light conditions in the Beijing laboratory on 5 March 2022, with soil moisture maintained (Figure A4). Geitonogamous pollination was performed at 7:00–10:00 on the flowering day [6], and pollination time was labeled. Anthers were divided into five developmental stages (Stages 1–5, detailed in Section 2.2), and anthers at each stage were collected. Ovaries at 4, 8, 12, and 20 DAP were also collected. Samples were immediately fixed in 2.5% glutaraldehyde fixative (pH 7.2–7.4) and cut into pieces <3 mm for subsequent use.

### 4.2. Observation of Morphological and Phenological Traits

Scape length of 15 individuals per form was measured daily from bud sprouting to elongation cessation. Initial/final flowering dates, initial/final fruit ripening dates were recorded, and flowering/fruiting periods were calculated as the number of days between initial and final dates. Total flower number per scape and mature capsule number were counted. Three healthy, uniform flowers and capsules were randomly selected daily, imaged with a Canon EOS 6D camera (Canon Inc.,Tokyo, Japan), and the long/short axes of 20 seeds and embryos per capsule were measured under a ZEISS stereomicroscope (Carl Zeiss AG, Oberkochen, Germany) until capsule maturation. Seed viability in mature dehiscing capsules was determined using 2% 2,3,5-triphenyltetrazolium chloride (TTC) staining (Solarbio, Beijing, China) [15]. Key indicators were calculated as follows:

(1) $$\mathrm{Scape}\;\mathrm{growth}\;\mathrm{rate}\;(\mathrm{cm}/\mathrm{day})\;=\;\frac{\left(Scape\;height\;at\;first\;flowering-Flower\;bud\;height\;at\;planting\right)}{Number\;of\;growth\;days}$$

(2) $$\mathrm{Ovary}\;\mathrm{expansion}\;\mathrm{rate}\;(\mathrm{mm}/\mathrm{day})=\frac{\left(Maximum\;ovary\;width\;at\;20\;DAP-Ovary\;width\;on\;pollination\;day\right)}{20}$$

(3) $$\mathrm{Seed}\;\mathrm{viability}\;(\%)=\frac{Number\;of\;viable\;seeds\times 100}{Total\;number\;of\;tested\;seeds}$$

### 4.3. Semi-Thin Section Preparation and Observation

**Figure A5:**
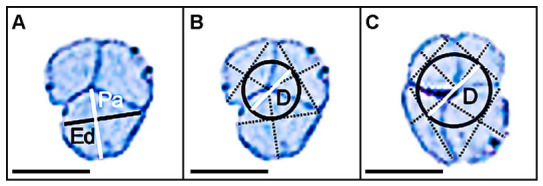
Measurements of pollen tetrad dimensions under light microscopy. (**A**) Size of a single pollen grain; Pa, polar axis; Ed, equatorial diameter. (**B**,**C**) Diameter measurements of pollen tetrads in two sectional planes; the diameter (D) of the circumscribed circle is defined by the intersections of polar axes and equatorial diameters of three (**B**) or four (**C**) individual pollen grains. Scale bar = 20 µm.

Semi-thin sections were prepared with minor modifications based on Li et al. [20]. Fixed samples were rinsed five times (10 min each) with 0.1 M phosphate buffer (pH 7.2), post-fixed with osmium tetroxide for 2 h, dehydrated in a graded acetone series (50%, 70%, 90%, 100% × 2, 15 min each), infiltrated with 50%, 70%, and pure Epon 812 resin (twice with pure resin, 8 h each), and polymerized at 65 °C for 24 h. Sections (70–250 nm) were cut with a Leica ultramicrotome (Leica Microsystems, Wetzlar, Germany), stained with 0.5% toluidine blue, and observed/photographed under a ZEISS optical microscope. Total anther locule wall thickness and endothecium thickness were measured before anther maturation. Polar axis (Pa), equatorial diameter (Ed) of single pollen grains, and tetrad diameter (*n* = 15) [21,70] were measured at full flowering; tetrad diameter was defined as the diameter of the circumscribed circle passing through cross-sections of three or four pollen grains (Figure A5) [71]. The proportion of compound pollen was counted at the elongation and separation stages: 10 non-overlapping fields of view were randomly selected per stage, and total pollen grains were counted (≥300 grains per stage). Tetrahedral, zygomorphic, and linear compound pollen were counted as one unit, and single pollen grains were fully dispersed individuals; damaged grains were excluded.

(4) $$\mathrm{Proportion}\;\mathrm{of}\;\mathrm{compound}\;\mathrm{pollen}\;(\%)=\frac{Total\;number\;of\;compound\;pollen\;units\times 100}{Total\;number\;of\;pollen\;units}$$

### 4.4. Ultra-Thin Sectioning and Transmission Electron Microscopy (TEM) Observation

Fixation, dehydration, infiltration, and embedding steps were the same as for semi-thin sections. Ultra-thin sections (70 nm) were cut, retrieved with copper grids, stained with uranyl acetate, and observed/photographed under a Hitachi HT7700 transmission electron microscope (Hitachi High-Technologies Corporation, Tokyo, Japan). Pollen wall stratification referenced the terminology of Halbritter et al. [29] and Purgina et al. [21]: Layer 1 (L1, exine outer layer) included the tectum, columellae, and foot layer; Layer 2 (L2) was the primexine before maturation and a distinct intermediate layer after maturation; Layer 3 (L3) was the innermost intine. The polar thickness ratio was measured as follows: locular-side thickness (L1 + L2 + L3) was the average of three regions with intact ornamentation, lateral wall thickness (L2 + L3) was the average of three uniform positions, and polar thickness ratio = Average locular-side total thickness/Average lateral wall total thickness [72].

(5) $$\mathrm{Polar}\;\mathrm{thickness}\;\mathrm{ratio}=\frac{Average\;locule-side\;total\;thickness}{Average\;lateral\;wall\;total\;thickness}$$

### 4.5. Statistical Analysis

Data were entered into Microsoft Excel 2019 (Microsoft Corporation, Redmond, WA, USA), statistically analyzed with IBM SPSS 26.0 (IBM Corp., Armonk, NY, USA), and plotted with GraphPad Prism 9 (GraphPad Software, San Diego, CA, USA). Morphological data were tested for normality and variance homogeneity (Levene test): One-Way ANOVA and Duncan’s post hoc test were used for homogeneous variances, while Welch’s ANOVA and Games–Howell post hoc test were used for heterogeneous variances. Data are presented as “mean ± SD”. Images were typeset and labeled with Adobe Photoshop CC 2019 (Adobe Inc., San Diego, CA, USA).

## 5. Conclusions

This study systematically compared the cytological differentiation characteristics of reproductive development in three *G. elata* forms via multi-timepoint dynamic observations. All forms follow a six-stage pollen wall development program characterized by “well-developed locular sides and simplified lateral sides”, with f.* elata* showing the highest sporopollenin deposition intensity, densest columellae, and fastest germination, while f. *glauca* exhibited delayed germination with no complete structures. We propose for the first time a potential two-step “chalazal degradation—micropylar localization” pattern in the female gametophyte, where vesicles may mediate the directional transport of degenerated nuclear debris, as indicated by vesicle clusters and fluid-like degradation zones. Direct experimental evidence is needed to confirm this hypothesis. Embryo development rate followed the order f. *elata* > f.* glauca* > f.* viridis*, with distinct resource allocation strategies: f. *elata* likely adopts a “fast-rhythm—high reserve” strategy with more embryo cells and high seed viability, f. *glauca* exhibits “multi-cell—low activity” traits, and f.* viridis* tends toward a “slow-development—stable quality” mode. Mature seeds are dominated by lipid and protein bodies, with f.* elata* uniquely containing amyloplasts and acicular phytin crystals; form-specific traits of seed coat radial wall protrusion length (f.* viridis* > f.* glauca* > f. *elata*) and inner tangential wall thickening morphology may adapt to wind dispersal and symbiotic germination. This study provides a theoretical basis for *G. elata* germplasm identification and protection, and offers new insights into reproductive adaptation of mycoheterotrophic Orchidaceae.

## Figures and Tables

**Table 1:** Classification of seed and embryo development in three *G. elata* forms based on morphological traits and growth dynamics (0–20 DAP).

Developmental Stage	Morphological Description	DAP (f.* glauca/*f. *elata/*f.* viridis*)	Seed Length Growth Phase	Embryo Growth Phase
I (Spherical stage)	Spherical seeds	0–1/0–3/0–2	Lag	Lag
II (Obovate stage)	Obovate seeds	2–3/4–6/3–5	Lag/Exponential	Lag
III (Elliptical stage)	Elliptical seeds	4–6/7–9/6–8	Exponential	Lag
IV (Fusiform initiation stage)	Fusiform seeds, embryo growth initiated	7–10/10/9	Exponential	Lag/Exponential
V (Embryo exponential growth stage)	Obovate embryos	11–15/11–14/10–15	Exponential/Stationary	Exponential
VI (Maturation stage)	Mature seeds and embryos	16–20/15–20/16–20	Stationary	Stationary

**Table 2:** Cytological stages and pollen wall developmental stages corresponding to anther developmental stages.

Anther Developmental Stage	*G. elata *f*. glauca*		*G. elata *f.* elata*		*G. elata *f.* viridis*	
	Cytological Stage	Pollen Wall Stage	Cytological Stage	Pollen Wall Stage	Cytological Stage	Pollen Wall Stage
Meristematic stage	1–3	I	1–3	I	1–3	I
Differentiation stage	3	I/II	4–5	III (late)	4	II/III (early-middle)
Elongation stage	5–6	IV (late)	5–6	IV (early-middle)	5–6	IV (late)
Separation stage (full anthesis)	6	V	6	ND	6	ND
Germination stage (8 h post-pollination)	ND	ND	germination	V/VI	germination	V/VI

Note: Cytological phases: 1 = Sporogenous cell phase; 2 = Microspore mother cell phase; 3 = Meiosis phase; 4 = Microspore determination phase; 5 = Mitosis phase; 6 = Mature pollen grain formation phase. Pollen wall developmental phases: I = Callose wall formation; II = Primexine formation; III = Sporopollenin accretion; IV = Callose wall degradation; V = Wall layer consolidation; VI = New pollen tube wall formation. ND = No detected.

**Table 3:** Comparative analysis of anther wall thickness and cytoplasmic channel width among three *G. elata* forms at different anther developmental stages.

Anther Developmental Stage	Form	Tapetal Degradation Status	Total Thickness of Anther Chamber Wall (μm, ***n*** = 4)		Thickness of Endothecium (μm, ***n*** = 4)		Width of Cytoplasmic Channel (μm, ***n*** = 20)
			Maximum	Minimum	Maximum	Minimum	
Meristematic stage	f.* glauca*	—	45.71 ± 4.95 ^a^	24.22 ± 0.66 ^a^	13.25 ± 0.25 ^b^	4.25 ± 1.17 ^a^	/
	f.* elata*	—	60.04 ± 10.02 ^a^	20.48 ± 2.12 ^a^	10.12 ± 0.45 ^b^	3.79 ± 0.76 ^a^	/
	f.* viridis*	—	83.37 ± 30.61 ^a^	47.7 ± 15.19 ^a^	23.24 ± 3.5 ^a^	6.51 ± 2.41 ^a^	/
Differentiation stage	f.* glauca*	—	95.09 ± 4.91 ^a^	42.61 ± 13.81 ^a^	34.89 ± 1.18 ^ab^	6.09 ± 2.21 ^b^	0.38 ± 0.14 ^b^
	f. *elata*	+	67.43 ± 5.45 ^b^	24.27 ± 1.89 ^a^	29.81 ± 0.85 ^b^	4.5 ± 0.95 ^b^	0.38 ± 0.14 ^a^
	f.* viridis*	—	90.77 ± 8.32 ^a^	58.32 ± 3.86 ^a^	39.81 ± 5.44 ^a^	17.82 ± 4.12 ^a^	0.49 ± 0.21 ^a^
Elongation stage	f. *glauca*	+	92.65 ± 13.6 ^a^	29.83 ± 9.92 ^a^	46.93 ± 4.82 ^a^	9.27 ± 2.62 ^a^	0.1 ± 0.06 ^b^
	f.* elata*	+	95.83 ± 7.4 ^a^	13.69 ± 1.86 ^a^	34.77 ± 1.48 ^b^	7 ± 1.32 ^a^	0.41 ± 0.12 ^a^
	f.* viridis*	+	87.91 ± 8.14 ^a^	20.4 ± 6.51 ^a^	40.34 ± 2.16 ^b^	7.98 ± 2.02 ^a^	0.49 ± 0.18 ^a^

Note: Values are presented as mean ± standard deviation (SD). The measurement criteria for anther wall thickness vary by developmental stage: meristematic stage = distance from epidermis to microspores; differentiation stage = distance from epidermis to inner middle layer; elongation stage = distance from epidermis to outer middle layer. “—” indicates no tapetal degradation; “+” indicates tapetal degradation; “/” indicates no cytoplasmic channels observed. Different lowercase letters indicate significant differences among forms (*p* < 0.05). For anther wall thickness (*n* = 4), measurements were taken from three anthers (three forms). For cytoplasmic channel width (*n* = 20), measurements were taken from the same three anthers (multiple channels per anther).

**Table 4:** Pollen wall thickness and polar ratio of three *G. elata* forms at different anther developmental stages (*n* = 20).

Anther Developmental Stage	Form	Locular-Side Primexine Thickness (μm)	Lateral-Side Primexine Thickness (μm)	Pollen Wall Thickness (μm)	Polar Ratio
Elongation stage	f. *glauca*	0.32–1.29	0.10–0.68	1.3 ± 0.2 ^aA^	4.8 ± 1.81 ^aA^
	f. *elata*	0.19–0.59	0.04–0.50	1.04 ± 0.23 ^b^	5.13 ± 1.49 ^a^
	f. *viridis*	0.18–1.51	0.16–0.71	1.03 ± 0.34 ^b^	2.91 ± 0.52 ^b^
Separation stage	f. *glauca*	—	—	1.39 ± 0.36 ^A^	2.4 ± 0.71 ^B^

Note: Values are presented as mean ± SD. Different lowercase letters indicate significant differences in the same index among forms at the elongation stage (*p* < 0.05); different uppercase letters indicate significant differences in the same index of *G. elata *f. *glauca* between development stages (*p* < 0.05). Pollen wall thickness refers to the average thickness of the locular-side pollen wall (distance from tectum to intine: L1 + L2 + L3). For pollen wall parameters (*n* = 20), measurements were obtained from three anthers (three forms), with 6–7 pollen grains measured per anther.

**Table 5:** Pollen grain dimensions of three *G. elata* forms.

Observation Indicators	*G. elata* f*. glauca*	*G. elata* f*. elata*	*G. elata *f.* viridis*
Tetrad diameter (μm)	15.61 ± 2.18 ^a^	11.74 ± 0.66 ^b^	12.36 ± 0.94 ^b^
Equatorial diameter (μm)	19.26 ± 2.27 ^a^	16.57 ± 1.96 ^b^	16.39 ± 1.26 ^b^
Polar axis (μm)	14.8 ± 1.83 ^a^	12.22 ± 1.9 ^b^	12.69 ± 1.42 ^b^

Note: *n* = 20 for tetrad diameter; *n* = 30 for equatorial diameter and polar axis. Values are presented as mean ± SD. Different lowercase letters indicate significant differences among forms (*p* < 0.05). All measurements were obtained from three anthers (three forms).

## Data Availability

The original contributions presented in this study are included in the article. The raw ultrastructural images, unprocessed measurement data, and experimental protocols are available from the corresponding author upon reasonable request. No restrictions apply to data access for non-commercial research purposes, provided that the original study is properly cited.

## Abbreviations

The following abbreviations are used in this manuscript:

A	Amyloplast
Ant	Anther
Bac	Baculae (sing. Baculum)
CW	Cell wall
Cyt	Cytoplasm
D	Diameter
DAP	Days after pollination
E	Epidermis
Ec	Egg cell
ECM	Ectomycorrhizal fungi
Ed	Equatorial diameter
EE	Embryo envelope
Em	Embryo
En	Endothecium
ER	Endoplasmic reticulum
FL	Foot layer
GA	Golgi apparatus
GC	Generative cell
GN	Generative nucleus
I	Intine
INF	Infratectum
LB	Lipid body
M	Mitochondrion
ML	Middle layer
MMC	Microspore mother cell
MS	Microspore
MVB	Multivesicular body
N	Nucleus
P	Plastid
Pa	Polar axis
PB	Protein body
PCD	Programmed cell death
PM	Plasma membrane
Pmx	Primexine
Pn	Polar nucleus
Prb	Probaculae (sing. Probaculum)
S	Stalk cell
SC	Seed coat
SD	Standard deviation
SG	Starch grain
Sy	Synergid
Ta	Tapetum
Tec	Tectum
TTC	2,3,5-triphenyltetrazolium chloride
V	Vacuole
VC	Vegetative cell
Ve	Secretory vesicle
VN	Vegetative nucleus

## Appendix A

**Table A1:** Morphological traits of flowers and capsules in three *G. elata* forms.

Form	Scape Growth Rate (cm/Day)	First Flowering Date	Flowering Duration (Days)	Flower Number per Plant	Perianth Tube Length (mm)	Perianth Tube Width (mm)	Ovary Expansion Rate (mm/Day)
f. *glauca*	1.75 ± 0.05 ^b^	Apr 26	9 ± 2 ^b^	62 ± 12 ^b^	8.69 ± 0.34 ^c^	6.52 ± 0.18 ^b^	0.21 ± 0.01 ^b^
f.* elata*	2.34 ± 0.22 ^a^	Apr 12	12 ± 2 ^a^	88 ± 21 ^a^	11.45 ± 0.48 ^a^	7.8 ± 0.22 ^a^	0.24 ± 0.02 ^a^
f. *viridis*	0.96 ± 0.05 ^c^	May 1	10 ± 2 ^b^	80 ± 8 ^a^	9.68 ± 0.32 ^b^	5.9 ± 0.08 ^c^	0.19 ± 0.02 ^c^
Form	Initial fruit ripening date	Fruiting period (days)	Capsule weight (g)	Capsule length (mm)	Capsule width (mm)	Capsule length-to-width ratio	S eed Viability (%)
f. *glauca*	May 15	17 ± 4 ^a^	0.4 ± 0.04 ^b^	25.32 ± 0.6 ^a^	8.51 ± 0.29 ^a^	2.98 ± 0.11 ^a^	56.54 ± 4.92 ^c^
f.* elata*	May 1	17 ± 3 ^a^	0.32 ± 0.03 ^c^	20.42 ± 0.74 ^b^	8.43 ± 0.29 ^a^	2.42 ± 0.13 ^b^	97.39 ± 2.96 ^a^
f. *viridis*	May 18	17 ± 2 ^a^	0.44 ± 0.03 ^a^	25.3 ± 0.56 ^a^	8.54 ± 0.22 ^a^	2.96 ± 0.1 ^a^	86.04 ± 5.64 ^b^

Note: *n* = 20 for flower and capsule size and seed viability; *n* = 15 for other characteristics. Values are presented as mean ± SD. Different lowercase letters within a column denote significant differences (*p* < 0.05) among forms.

**Table A2:** Core ultrastructural parameters of mature seeds (20 DAP) among three *G. elata* forms.

Structural Parameters	*G. elata *f*. glauca*	*G. elata* f. *elata*	*G. elata* f. *viridis*
Seed coat membrane thickness range (μm)	0.37–1.88	0.19–0.66	0.18–1.86
Lipid body diameter range in embryo cells (μm)	0.43–3.72	0.35–5.90	0.28–4.74
Maximum thickness of stalk cell wall (μm)	2.16–4.56	1.45–1.63	1.48
Seed coat radial wall protrusion length range (μm)	13.67–45.78	8.44–17.49	11.18–46.11
Seed coat inner tangential wall thickening pattern	Continuously slightly undulate	Triangular-serrate	Continuously undulate
Storage material characteristics	Lipid bodies; polymorphic protein bodies (no amyloplasts)	Lipid bodies; a few amyloplasts; acicular crystals	Lipid bodies; protein bodies (no amyloplasts)

**Table A3:** Temporal developmental characteristics of embryo and integument tissues among three *G. elata* forms.

DAP	Developmental Traits	*G. elata *f.* glauca*	*G. elata* f.* elata*	*G. elata *f. *viridis*
4	Integument cell degradation	Undegraded (containing large starch grains)	Undegraded (containing large starch grains)	Undegraded (organelle-rich cytoplasm)
	Embryo membrane formation	Not initiated	Not initiated	Not initiated
	Suspensor cells	Not formed (zygote stage)	Formed (functionally active)	Not formed (fertilization in progress)
	Embryo body	Zygote	Zygote (nucellar epidermis cell degeneration)	Zygote not observed (polar nucleus fusion in progress)
	Embryo-periphery relationship	Direct contact with integument cytoplasm	Direct contact with integument cytoplasm	—
8	Integument cell degradation	Initiation stage (cytoplasm release + naked nuclei)	Initiation stage (cytoplasm release + naked nuclei)	Initiation stage (cytoplasm degradation)
	Embryo membrane formation	Not initiated	Not initiated	Not initiated
	Suspensor cells	Forming	Intact (large vacuolization)	Presumed to exist (functionally active)
	Embryo body	2-celled proembryo	2–5-celled proembryo	5-celled embryo section
	Embryo-periphery relationship	Direct contact with integument cytoplasm	Direct contact with integument cytoplasm	Contact with degraded integument cytoplasm
12	Integument cell degradation	Progressive stage (cytoplasmic layer retained)	Progressive stage (cytoplasmic layer retained)	Progressive stage (cytoplasmic layer retained)
	Embryo membrane formation	Establishing (flocculent material forming loops)	Establishing (flocculent material forming loops)	Establishing (flocculent material forming loops)
	Suspensor cells	Not observed (presumed degraded)	Degrading (nuclear disintegration)	Degrading (remnant structure)
	Embryo body	4–20 cells (starch-rich)	2–22 cells (including 18–20 cell intact proembryo)	8–14 cells (unicellular section included)
	Embryo-periphery relationship	Forming embryo membrane, surrounded by integument cytoplasm	Forming embryo membrane, surrounded by integument cytoplasm	Forming embryo membrane, surrounded by integument cytoplasm
20	Integument cell degradation	Completion stage (no cytoplasmic layer)	Completion stage (no cytoplasmic layer)	Completion stage (no cytoplasmic layer)
	Embryo membrane formation	Fully formed (clear dark black edge)	Fully formed (clear dark black edge)	Fully formed (clear dark black edge)
	Suspensor cells	Completely degenerated (only thickened cell wall remains)	Completely degenerated (only thickened cell wall remains)	Completely degenerated (only thickened cell wall remains)
	Embryo body	21 cells (mature and independent)	23–31 cells (mature and independent)	7–10 cells (mature and independent)
	Embryo-periphery relationship	Cavity exists between embryo membrane and seed coat	Cavity exists between embryo membrane and seed coat	Cavity exists between embryo membrane and seed coat

Note: “—” indicates that zygote/embryo formation has not yet occurred in *G. elata* f. *viridis* at 4 DAP; thus, no embryo–periphery relationship can be recorded.
